# Febuxostat methanol solvate

**DOI:** 10.1107/S1600536811014905

**Published:** 2011-04-29

**Authors:** Qi-Ying Jiang, Jing-Jing Qian, Jian-Ming Gu, Gu-Ping Tang, Xiu-Rong Hu

**Affiliations:** a Institute of Chemical Biology and Pharmaceutical Chemistry, Zhejiang University, Hangzhou, Zhejiang 310028, People’s Republic of China; b College of Pharmaceutical Science, Zhejiang Chinese Medical University, Hangzhou, Zhejiang 310053, People’s Republic of China; c Center of Analysis and Measurement, Zhejiang University, Hangzhou, Zhejiang 310028, People’s Republic of China

## Abstract

In the title compound {systematic name: [2-(3-cyano-4-isobutyl­oxyphen­yl)-4-methyl-1,3-thia­zole-5-carb­oxy­lic acid (febuxostat) methanol monosolvate}, C_16_H_16_N_2_O_3_S·CH_4_O, the benzene and thia­zole rings in the febuxostat mol­ecule are twisted at 5.3 (1)°. In the crystal structure, inter­molecular O—H⋯O and O—H⋯N hydrogen bonds link the febuxostat and methanol mol­ecules into helical chains along the 2_1_ screw axis.

## Related literature

For applications of febuxostat in the medicine, see: Schumacher *et al.* (2009 ▶); Becke *et al.* (2010 ▶); Khosravan *et al.* (2007 ▶); Takano *et al.* (2005 ▶). For the synthesis, polymorphism, stability and bioavailabitily of febuxostat, see: Hiramatsu *et al.* (2000 ▶); Sorbera *et al.* (2001 ▶); Zhou *et al.* (2007 ▶). For the crystal structure of febuxostat pyridine solvate, see: Zhu *et al.* (2009 ▶).
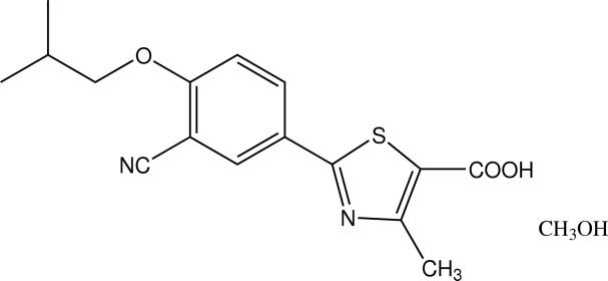


## Experimental

### 

#### Crystal data

C_16_H_16_N_2_O_3_S·CH_4_O
*M* *_r_* = 348.41
Monoclinic, 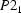

*a* = 4.7089 (3) Å
*b* = 17.9073 (13) Å
*c* = 10.7965 (8) Å
β = 98.047 (2)°
*V* = 901.44 (11) Å^3^
*Z* = 2
Mo *K*α radiation
μ = 0.20 mm^−1^
*T* = 296 K
0.48 × 0.13 × 0.10 mm

#### Data collection

Rigaku R-AXIS RAPID/ZJUG diffractometer
Absorption correction: multi-scan (*ABSCOR*; Higashi, 1995 ▶) *T*_min_ = 0.905, *T*_max_ = 0.980
8429 measured reflections
4048 independent reflections
3048 reflections with *I* > 2σ(*I*)
*R*_int_ = 0.034

#### Refinement

*R*[*F* ^2^ > 2σ(*F* ^2^)] = 0.043
*wR*(*F* ^2^) = 0.078
*S* = 1.00
4048 reflections
223 parameters
1 restraint
H-atom parameters constrained
Δρ_max_ = 0.13 e Å^−3^
Δρ_min_ = −0.15 e Å^−3^
Absolute structure: Flack (1983 ▶), with 1941 Friedel pairs
Flack parameter: −0.05 (7)

### 

Data collection: *PROCESS-AUTO* (Rigaku, 2006 ▶); cell refinement: *PROCESS-AUTO*; data reduction: *CrystalStructure* (Rigaku, 2007 ▶); program(s) used to solve structure: *SHELXS97* (Sheldrick, 2008 ▶); program(s) used to refine structure: *SHELXL97* (Sheldrick, 2008 ▶); molecular graphics: *ORTEP-3 for Windows* (Farrugia, 1997 ▶); software used to prepare material for publication: *WinGX* (Farrugia, 1999 ▶).

## Supplementary Material

Crystal structure: contains datablocks global, I. DOI: 10.1107/S1600536811014905/cv5075sup1.cif

Structure factors: contains datablocks I. DOI: 10.1107/S1600536811014905/cv5075Isup2.hkl

Supplementary material file. DOI: 10.1107/S1600536811014905/cv5075Isup3.cml

Additional supplementary materials:  crystallographic information; 3D view; checkCIF report

## Figures and Tables

**Table 1:** Hydrogen-bond geometry (Å, °)

*D*—H⋯*A*	*D*—H	H⋯*A*	*D*⋯*A*	*D*—H⋯*A*
O4—H4⋯N1^i^	0.82	2.09	2.899 (3)	169
O1—H1⋯O4	0.82	1.80	2.608 (3)	166

Symmetry code: (i) 
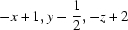
.

**Fig. 1.**
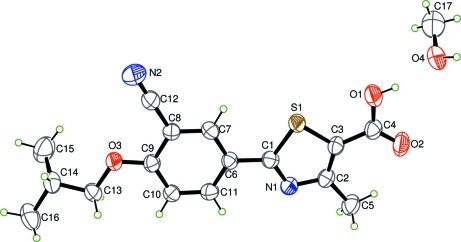
Molecular structure of (I) showing the atom-labelling scheme and displacement ellipsoids drawn at 40% probability level. H atoms are shown as small circles of arbitary radii.

**Fig. 2.**
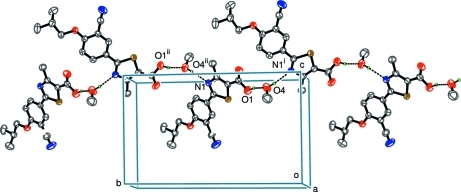
A view down the c axis of a portion of the crystal structure showing hydrogen bonds by dashed lines [symmetry codes: (i) 1 - x, -1/2 + y, 2 - z; (ii) 1 - x, 1/2 + y, 2 - z].

## supplementary crystallographic information

### Comment

Gout is a disorder caused by deposition of monosodium urate crystals in joints and other tissues as a result of extracellular urate supersaturation. However, hyperuricemia is the most important risk factor for the development of gout and occurs as a result of increased uric acid production (Takano *et al.*, 2005). Febuxostat is a novel non-purine selective inhibitor of xanthine oxidase which is currently under investigation for the management of hyperuricaemia in patients with gout (Khosravan *et al.*, 2007). Many patents and papers have been reported on the synthesis, polymorphism, stability and bioavailabitily of this drug (Hiramatsu *et al.*, 2000; Sorbera *et al.*, 2001; Zhou *et al.*, 2007). The single-crystal structure of febuxostat pyridine solvate has been reported by Zhu *et al.* (2009). In the present study, we report the crystal structure of febuxostat methanol solvate (I).

The asymmetric unit of (I) consists of one febuxostat molecule and one methanol molecule (Fig. 1). The benzene and thiazole rings of the febuxostat molecule are almost coplanar, with the dihedral angle between them being 5.3 (1)°. The carboxyl group is coplanar with the thiazole ring as indicated by torsion angles O2—C4—C3—C2 and O1—C4—C3—S1 of -0.8 (4)° and -2.5 (3)°, respectively. Conformations of the febuxostat molecule in (I) and in febuxostat pyridine solvate (Zhu *et al.*, 2009) are close.

In the crystal structure, febuxostat molecules and methanol molecule are linked by intermolecular hydrogen bonds O—H···N and O—H···O. In this way, the molecules are linked into infinite zigzag chains stretching along the *b* axis.

### Experimental

The crude product supplied by Zhejiang Huadong Pharmaceutical Co., Ltd, was recrystallized from methanol solution giving colourless crystals suitable for X-ray diffraction.

### Refinement

All H atoms were placed in calculated positions with C—H = 0.93–0.98 Å and O—H = 0.82 Å and included in the refinement in riding model, with *U*_iso_(H) = 1.2*U*_eq_ or 1.5*U*_eq_(carrier atom).

### Crystal data

**Table:**

C_16_H_16_N_2_O_3_S·CH_4_O	*F*(000) = 368
*M**_r_* = 348.41	*D*_x_ = 1.284 Mg m^−^^3^
Monoclinic, *P*2_1_	Mo *K*α radiation, λ = 0.71073 Å
Hall symbol: P 2yb	Cell parameters from 6196 reflections
*a* = 4.7089 (3) Å	θ = 3.8–27.4°
*b* = 17.9073 (13) Å	µ = 0.20 mm^−^^1^
*c* = 10.7965 (8) Å	*T* = 296 K
β = 98.047 (2)°	Needle, colourless
*V* = 901.44 (11) Å^3^	0.48 × 0.13 × 0.10 mm
*Z* = 2	

### Data collection

**Table:**

Rigaku R-AXIS RAPID/ZJUG diffractometer	4048 independent reflections
Radiation source: rolling anode	3048 reflections with *I* > 2σ(*I*)
graphite	*R*_int_ = 0.034
Detector resolution: 10.00 pixels mm^-1^	θ_max_ = 27.4°, θ_min_ = 3.8°
ω scans	*h* = −5→6
Absorption correction: multi-scan (*ABSCOR*; Higashi, 1995)	*k* = −23→23
*T*_min_ = 0.905, *T*_max_ = 0.980	*l* = −13→13
8429 measured reflections	

### Refinement

**Table:**

Refinement on *F*^2^	Secondary atom site location: difference Fourier map
Least-squares matrix: full	Hydrogen site location: inferred from neighbouring sites
*R*[*F*^2^ > 2σ(*F*^2^)] = 0.043	H-atom parameters constrained
*wR*(*F*^2^) = 0.078	*w* = 1/[σ^2^(*F*_o_^2^) + (0.0221*P*)^2^ + 0.134*P*] where *P* = (*F*_o_^2^ + 2*F*_c_^2^)/3
*S* = 1.00	(Δ/σ)_max_ = 0.002
4048 reflections	Δρ_max_ = 0.13 e Å^−^^3^
223 parameters	Δρ_min_ = −0.15 e Å^−^^3^
1 restraint	Absolute structure: Flack (1983), with 1941 Friedel pairs
Primary atom site location: structure-invariant direct methods	Flack parameter: −0.05 (7)

### Special details

**Table:**

Geometry. All e.s.d.'s (except the e.s.d. in the dihedral angle between two l.s. planes) are estimated using the full covariance matrix. The cell e.s.d.'s are taken into account individually in the estimation of e.s.d.'s in distances, angles and torsion angles; correlations between e.s.d.'s in cell parameters are only used when they are defined by crystal symmetry. An approximate (isotropic) treatment of cell e.s.d.'s is used for estimating e.s.d.'s involving l.s. planes.
Refinement. Refinement of *F*^2^ against ALL reflections. The weighted *R*-factor *wR* and goodness of fit *S* are based on *F*^2^, conventional *R*-factors *R* are based on *F*, with *F* set to zero for negative *F*^2^. The threshold expression of *F*^2^ > σ(*F*^2^) is used only for calculating *R*-factors(gt) *etc*. and is not relevant to the choice of reflections for refinement. *R*-factors based on *F*^2^ are statistically about twice as large as those based on *F*, and *R*-factors based on ALL data will be even larger.

### Fractional atomic coordinates and isotropic or equivalent isotropic displacement parameters (Å^2^)

**Table:**

	*x*	*y*	*z*	*U*_iso_*/*U*_eq_	
O4	0.2078 (5)	0.19681 (11)	0.8788 (2)	0.0794 (6)	
H4	0.2298	0.1575	0.9181	0.119*	
C17	0.0129 (8)	0.1856 (2)	0.7690 (3)	0.0918 (10)	
H17A	−0.1528	0.1595	0.7890	0.138*	
H17B	0.1031	0.1567	0.7106	0.138*	
H17C	−0.0445	0.2331	0.7324	0.138*	
S1	0.81600 (13)	0.45188 (3)	0.82299 (5)	0.04563 (15)	
N1	0.7895 (4)	0.56137 (10)	0.97388 (17)	0.0410 (4)	
O3	1.6085 (4)	0.72442 (8)	0.62269 (15)	0.0491 (4)	
C6	1.0840 (5)	0.59027 (12)	0.8089 (2)	0.0377 (5)	
C8	1.3569 (5)	0.61374 (13)	0.6395 (2)	0.0411 (6)	
C1	0.8996 (5)	0.54145 (12)	0.8730 (2)	0.0403 (5)	
C13	1.6920 (5)	0.79823 (12)	0.6671 (2)	0.0465 (6)	
H13A	1.8109	0.7952	0.7479	0.056*	
H13B	1.5232	0.8276	0.6764	0.056*	
O1	0.5033 (5)	0.31992 (10)	0.87756 (19)	0.0708 (6)	
H1	0.4149	0.2820	0.8907	0.106*	
C3	0.6208 (5)	0.44077 (13)	0.9457 (2)	0.0420 (6)	
C14	1.8573 (6)	0.83447 (13)	0.5728 (2)	0.0504 (6)	
H14	2.0217	0.8026	0.5625	0.060*	
C2	0.6304 (5)	0.50420 (13)	1.0150 (2)	0.0416 (5)	
C11	1.1728 (5)	0.65996 (13)	0.8577 (2)	0.0463 (6)	
H11	1.1123	0.6757	0.9318	0.056*	
O2	0.3404 (4)	0.35831 (10)	1.05077 (18)	0.0692 (5)	
C9	1.4399 (5)	0.68379 (13)	0.6888 (2)	0.0406 (5)	
C7	1.1807 (5)	0.56763 (12)	0.6989 (2)	0.0443 (6)	
H7	1.1269	0.5212	0.6648	0.053*	
C10	1.3472 (5)	0.70618 (13)	0.7997 (2)	0.0453 (6)	
H10	1.4029	0.7523	0.8346	0.054*	
C4	0.4753 (5)	0.36987 (14)	0.9650 (2)	0.0480 (6)	
C12	1.4636 (6)	0.58932 (14)	0.5282 (3)	0.0572 (7)	
C5	0.4890 (6)	0.51762 (15)	1.1292 (2)	0.0568 (7)	
H5A	0.6307	0.5160	1.2023	0.085*	
H5B	0.3475	0.4797	1.1352	0.085*	
H5C	0.3982	0.5657	1.1233	0.085*	
C16	1.9705 (7)	0.90956 (14)	0.6279 (3)	0.0750 (9)	
H16A	2.0798	0.9015	0.7087	0.113*	
H16B	1.8119	0.9420	0.6359	0.113*	
H16C	2.0906	0.9321	0.5734	0.113*	
N2	1.5505 (7)	0.56898 (16)	0.4403 (3)	0.0918 (9)	
C15	1.6760 (7)	0.8435 (2)	0.4470 (3)	0.0838 (10)	
H15A	1.5222	0.8777	0.4543	0.126*	
H15B	1.5982	0.7959	0.4189	0.126*	
H15C	1.7922	0.8626	0.3879	0.126*	

### Atomic displacement parameters (Å^2^)

**Table:**

	*U*^11^	*U*^22^	*U*^33^	*U*^12^	*U*^13^	*U*^23^
O4	0.1106 (17)	0.0497 (12)	0.0779 (14)	−0.0241 (12)	0.0131 (14)	0.0098 (11)
C17	0.102 (3)	0.086 (2)	0.088 (2)	−0.013 (2)	0.017 (2)	0.012 (2)
S1	0.0576 (3)	0.0349 (3)	0.0467 (3)	−0.0026 (3)	0.0151 (3)	−0.0007 (3)
N1	0.0452 (10)	0.0372 (10)	0.0415 (10)	0.0046 (9)	0.0089 (9)	0.0009 (8)
O3	0.0636 (11)	0.0400 (9)	0.0473 (9)	−0.0117 (8)	0.0204 (9)	−0.0047 (7)
C6	0.0410 (12)	0.0334 (11)	0.0389 (12)	0.0016 (10)	0.0058 (10)	0.0038 (10)
C8	0.0465 (14)	0.0380 (12)	0.0399 (13)	−0.0025 (11)	0.0101 (11)	−0.0030 (10)
C1	0.0435 (13)	0.0358 (12)	0.0407 (12)	0.0018 (10)	0.0029 (11)	0.0024 (10)
C13	0.0519 (14)	0.0375 (12)	0.0509 (14)	−0.0060 (12)	0.0101 (12)	−0.0024 (11)
O1	0.1015 (16)	0.0449 (11)	0.0727 (13)	−0.0199 (10)	0.0356 (12)	−0.0043 (10)
C3	0.0475 (13)	0.0388 (14)	0.0408 (12)	0.0016 (11)	0.0097 (10)	0.0076 (11)
C14	0.0499 (14)	0.0417 (14)	0.0627 (16)	−0.0004 (11)	0.0194 (13)	0.0036 (12)
C2	0.0424 (14)	0.0426 (13)	0.0402 (13)	0.0074 (11)	0.0069 (11)	0.0075 (10)
C11	0.0565 (15)	0.0422 (14)	0.0407 (13)	−0.0020 (12)	0.0089 (12)	−0.0045 (11)
O2	0.0964 (15)	0.0531 (11)	0.0647 (12)	−0.0073 (10)	0.0341 (12)	0.0151 (9)
C9	0.0456 (13)	0.0357 (12)	0.0408 (13)	−0.0004 (10)	0.0072 (11)	0.0025 (10)
C7	0.0503 (13)	0.0351 (12)	0.0473 (13)	−0.0029 (11)	0.0062 (11)	−0.0024 (11)
C10	0.0595 (16)	0.0345 (12)	0.0427 (13)	−0.0059 (11)	0.0105 (12)	−0.0027 (10)
C4	0.0562 (15)	0.0423 (13)	0.0452 (14)	0.0044 (12)	0.0057 (13)	0.0100 (12)
C12	0.0730 (18)	0.0450 (14)	0.0577 (16)	−0.0140 (13)	0.0229 (14)	−0.0090 (13)
C5	0.0666 (17)	0.0557 (16)	0.0531 (16)	0.0009 (13)	0.0252 (14)	0.0004 (12)
C16	0.074 (2)	0.0436 (16)	0.113 (3)	−0.0099 (14)	0.0326 (19)	−0.0034 (16)
N2	0.126 (2)	0.085 (2)	0.0752 (18)	−0.0245 (18)	0.0498 (18)	−0.0323 (16)
C15	0.100 (3)	0.087 (2)	0.067 (2)	−0.004 (2)	0.0197 (19)	0.0235 (17)

### Geometric parameters (Å, °)

**Table:**

O4—C17	1.409 (4)	C3—C2	1.357 (3)
O4—H4	0.8200	C3—C4	1.471 (3)
C17—H17A	0.9600	C14—C15	1.508 (4)
C17—H17B	0.9600	C14—C16	1.535 (4)
C17—H17C	0.9600	C14—H14	0.9800
S1—C1	1.720 (2)	C2—C5	1.501 (3)
S1—C3	1.727 (2)	C11—C10	1.377 (3)
N1—C1	1.319 (3)	C11—H11	0.9300
N1—C2	1.378 (3)	O2—C4	1.211 (3)
O3—C9	1.352 (3)	C9—C10	1.389 (3)
O3—C13	1.442 (3)	C7—H7	0.9300
C6—C7	1.390 (3)	C10—H10	0.9300
C6—C11	1.396 (3)	C12—N2	1.144 (3)
C6—C1	1.472 (3)	C5—H5A	0.9600
C8—C7	1.389 (3)	C5—H5B	0.9600
C8—C9	1.397 (3)	C5—H5C	0.9600
C8—C12	1.435 (4)	C16—H16A	0.9600
C13—C14	1.512 (3)	C16—H16B	0.9600
C13—H13A	0.9700	C16—H16C	0.9600
C13—H13B	0.9700	C15—H15A	0.9600
O1—C4	1.321 (3)	C15—H15B	0.9600
O1—H1	0.8200	C15—H15C	0.9600
			
C17—O4—H4	109.5	C3—C2—C5	127.0 (2)
O4—C17—H17A	109.5	N1—C2—C5	118.0 (2)
O4—C17—H17B	109.5	C10—C11—C6	122.1 (2)
H17A—C17—H17B	109.5	C10—C11—H11	118.9
O4—C17—H17C	109.5	C6—C11—H11	118.9
H17A—C17—H17C	109.5	O3—C9—C10	125.3 (2)
H17B—C17—H17C	109.5	O3—C9—C8	115.8 (2)
C1—S1—C3	89.40 (12)	C10—C9—C8	118.8 (2)
C1—N1—C2	111.03 (19)	C6—C7—C8	120.6 (2)
C9—O3—C13	118.06 (17)	C6—C7—H7	119.7
C7—C6—C11	117.8 (2)	C8—C7—H7	119.7
C7—C6—C1	120.9 (2)	C11—C10—C9	119.9 (2)
C11—C6—C1	121.3 (2)	C11—C10—H10	120.1
C7—C8—C9	120.8 (2)	C9—C10—H10	120.1
C7—C8—C12	120.5 (2)	O2—C4—O1	123.1 (2)
C9—C8—C12	118.7 (2)	O2—C4—C3	124.1 (2)
N1—C1—C6	123.6 (2)	O1—C4—C3	112.8 (2)
N1—C1—S1	114.35 (17)	N2—C12—C8	179.0 (3)
C6—C1—S1	122.03 (18)	C2—C5—H5A	109.5
O3—C13—C14	108.14 (19)	C2—C5—H5B	109.5
O3—C13—H13A	110.1	H5A—C5—H5B	109.5
C14—C13—H13A	110.1	C2—C5—H5C	109.5
O3—C13—H13B	110.1	H5A—C5—H5C	109.5
C14—C13—H13B	110.1	H5B—C5—H5C	109.5
H13A—C13—H13B	108.4	C14—C16—H16A	109.5
C4—O1—H1	109.5	C14—C16—H16B	109.5
C2—C3—C4	128.6 (2)	H16A—C16—H16B	109.5
C2—C3—S1	110.26 (18)	C14—C16—H16C	109.5
C4—C3—S1	121.10 (18)	H16A—C16—H16C	109.5
C15—C14—C13	111.9 (2)	H16B—C16—H16C	109.5
C15—C14—C16	112.2 (2)	C14—C15—H15A	109.5
C13—C14—C16	107.4 (2)	C14—C15—H15B	109.5
C15—C14—H14	108.4	H15A—C15—H15B	109.5
C13—C14—H14	108.4	C14—C15—H15C	109.5
C16—C14—H14	108.4	H15A—C15—H15C	109.5
C3—C2—N1	115.0 (2)	H15B—C15—H15C	109.5
			
C2—N1—C1—C6	−178.87 (19)	C7—C6—C11—C10	0.9 (3)
C2—N1—C1—S1	−0.2 (2)	C1—C6—C11—C10	179.5 (2)
C7—C6—C1—N1	−176.4 (2)	C13—O3—C9—C10	−2.4 (3)
C11—C6—C1—N1	5.0 (3)	C13—O3—C9—C8	177.84 (19)
C7—C6—C1—S1	5.0 (3)	C7—C8—C9—O3	−179.2 (2)
C11—C6—C1—S1	−173.57 (17)	C12—C8—C9—O3	2.6 (3)
C3—S1—C1—N1	0.00 (18)	C7—C8—C9—C10	1.1 (3)
C3—S1—C1—C6	178.72 (18)	C12—C8—C9—C10	−177.2 (2)
C9—O3—C13—C14	−175.41 (19)	C11—C6—C7—C8	−0.8 (3)
C1—S1—C3—C2	0.17 (18)	C1—C6—C7—C8	−179.4 (2)
C1—S1—C3—C4	−179.20 (18)	C9—C8—C7—C6	−0.2 (3)
O3—C13—C14—C15	62.2 (3)	C12—C8—C7—C6	178.1 (2)
O3—C13—C14—C16	−174.3 (2)	C6—C11—C10—C9	0.1 (4)
C4—C3—C2—N1	179.0 (2)	O3—C9—C10—C11	179.3 (2)
S1—C3—C2—N1	−0.3 (2)	C8—C9—C10—C11	−1.0 (3)
C4—C3—C2—C5	−0.8 (4)	C2—C3—C4—O2	−0.8 (4)
S1—C3—C2—C5	179.91 (19)	S1—C3—C4—O2	178.45 (19)
C1—N1—C2—C3	0.3 (3)	C2—C3—C4—O1	178.3 (2)
C1—N1—C2—C5	−179.9 (2)	S1—C3—C4—O1	−2.5 (3)

### Hydrogen-bond geometry (Å, °)

**Table:**

*D*—H···*A*	*D*—H	H···*A*	*D*···*A*	*D*—H···*A*
O4—H4···N1^i^	0.82	2.09	2.899 (3)	169
O1—H1···O4	0.82	1.80	2.608 (3)	166

Symmetry codes: (i) −*x*+1, *y*−1/2, −*z*+2.

## References

[bb1] Becke, M. A., Schumacher, H. R., Espinoza, L. R., Wells, A. F., Macdonald, P., Lloyd, E. & Lademacher, C. (2010). *Arthritis Res. Ther.* **12**, R63, 1–12. 10.1186/ar2978 PMC2888216 20370912

[bb2] Farrugia, L. J. (1997). *J. Appl. Cryst.* **30**, 565.

[bb3] Farrugia, L. J. (1999). *J. Appl. Cryst.* **32**, 837–838.

[bb4] Flack, H. D. (1983). *Acta Cryst.* A**39**, 876–881.

[bb5] Higashi, T. (1995). *ABSCOR* Rigaku Corporation, Tokyo, Japan.

[bb6] Hiramatsu, T., Matsumoto, K. & Watanabe, K. (2000). China Patent CN 1275126.

[bb7] Khosravan, R., Grabowski, B., Wu, J. T., Joseph-Ridge, N. & Vernillet, L. (2007). *Clin. Pharm.* **65**, 355–363. 10.1111/j.1365-2125.2007.03016.x PMC2291255 17953718

[bb8] Rigaku (2006). *PROCESS-AUTO* Rigaku Corporation, Tokyo, Japan.

[bb9] Rigaku (2007). *CrystalStructure* Rigaku Corporation, Tokyo, Japan.

[bb10] Schumacher, H. R., Becker, M. A., Lloyd, E., Macdonald, P. A. & Lademacher, C. (2009). *Rheumatology*, **48**, 188–194. 10.1093/rheumatology/ken457 19141576

[bb11] Sheldrick, G. M. (2008). *Acta Cryst.* A**64**, 112–122. 10.1107/S0108767307043930 18156677

[bb12] Sorbera, L. A., Revel, L., Rabasseda, X. & Castaner, J. (2001). *Drugs Fut.* **26**, 32–38.

[bb13] Takano, Y., Hase-Aoki, K., Horiuchi, H., Zhao, L., Kasahara, Y., Kondo, S. & Becker, M. A. (2005). *Life Sci.* **76**, 1835–1847. 10.1016/j.lfs.2004.10.031 15698861

[bb14] Zhou, X. G., Tang, X. M., Deng, J., Ye, W. R., Luo, J., Zhang, D. L. & Fan, B. (2007). China Patent CN 1970547.

[bb15] Zhu, X., Wang, Y. & Lu, T. (2009). *Acta Cryst.* E**65**, o2603. 10.1107/S1600536809039002 PMC2971045 21578222

